# Translation, cross-cultural adaptation and psychometric properties of the Arabic version of the Fremantle Knee Awareness Questionnaire in people with knee osteoarthritis

**DOI:** 10.1371/journal.pone.0328228

**Published:** 2025-07-15

**Authors:** Mohammad Madi, Hayat Hamzeh, Sumayeh Abujaber, Ibrahim Altubasi

**Affiliations:** 1 Department of Physiotherapy, Faculty of Applied Medical Sciences, The Hashemite University, Zarqa, Jordan; 2 Department of Physiotherapy, School of Rehabilitation Sciences, The University of Jordan, Amman, Jordan; 3 Leeds institute of Medical Research, University of Leeds, Leeds, United Kingdom; Iran University of Medical Sciences, IRAN, ISLAMIC REPUBLIC OF

## Abstract

**Background:**

The presence of distorted body awareness that is associated with knee osteoarthritis (OA) is increasingly reported in the literature. The Fremantle Knee Awareness Questionnaire (FreKAQ) is a self-reported nine-item questionnaire developed to measure distorted awareness of the knee joint. No Arabic language version of FreKAQ is currently available.

**Objective:**

The study’s first aim was to cross-culturally adapt FreKAQ into the Arabic language. The second aim was to test the psychometric properties of the adapted Arabic version (FreKAQ-A).

**Methods:**

The adaptation phase was conducted according to recommended guidelines for translation and cultural adaptation of self-reported outcome measures. The second phase of the study evaluated the internal consistency, test-retest reliability, and validity of FreKAQ-A. Cronbach’s alpha and Intraclass Correlation Coefficient (ICC) were used to test the scales’ internal consistency and test-retest reliability, respectively. Validity was tested with six pre-specified hypotheses. Pain Catastrophizing Scale (PCS), Health-related Quality of Life scales (EQ-5D), Central Sensitization Inventory (CSI), and the Chronic Pain Self-efficacy Scale (CPSS) were used.

**Results:**

After successful adaptation, individuals with osteoarthritic knee pain were recruited. A total of 197 individuals (19.7% males) completed the first round of reliability measurement. Almost 60% had pain in both knees. FreKAQ-A showed excellent degree of internal consistency (Cronbach’s α = 0.87). The data provided strong support to five out of the six pre-specified discriminative validity hypotheses. There was a strong correlation between FreKAQ-A and PCS (*rho *= 0.71) and EQ-5D-5L (*rho *= 0.62). Seventy individuals participated in the second round of reliability measurement. Intraclass correlation coefficient was excellent (ICC_2,1_ = 0.841).

**Conclusion:**

The study provides the first Arabic adaptation of FreKAQ. The adapted version has excellent reliability and validity. It can be used for individuals with knee osteoarthritis to explore the presence of distorted body awareness, leading to tailored management in cases with persistent knee pain.

## Introduction

Knee osteoarthritis (OA) is a degenerative chronic condition with a global prevalence of 22 ⋅ 9% in older adults [1]. Its prevalence in Arabic-speaking countries has increased 2.88-fold between 1990 and 2019 [2]. While this varies between individual countries, it is generally higher compared to other populations [3].

Distorted awareness of the knee has been recently identified in many patients with persistent OA related knee pain [4,5]. Symptoms such as neglect-like symptoms (e.g., impaired attention or awareness), decreased proprioceptive acuity (e.g., recognition of knee position), and distorted body image (e.g., sense of enlarged knee) have all been reported [6]. Clinical recognition of these symptoms can lead to targeted and personalised interventions such as cognitive therapies, neuromuscular coordination and proprioception training, and visual feedback therapy, respectively. Notably, treatment techniques that provide visual and tactile illusions to restore knee joint awareness have been shown to decrease pain [7,8].

Recent evidence suggests that distorted awareness can lead to persistent pain and disability regardless of the degree of degenerative changes in the joint, which implies an involvement of the central nervous system [9,10,11]. Although the mechanism that leads to this is not fully understood, changes in the cortical representation of the affected area has been identified in individuals with knee OA [12], and it is thought to directly lead to the altered perception [13]. Additionally, psychological factors such as anxiety and depression which are common in individuals with chronic pain are thought to indirectly lead to difficulties in accurate perception of the body [14,15]. Further, changes within sensory organs and afferent fibres including nociceptors, which are identified in multiple chronic pain conditions, might lead to altered transmission of signal and subsequent disturbance in body awareness [16–19].

Distorted body perception with chronic painful conditions extends beyond the knee joint. It has been identified in other body parts such as the back [20], shoulders [21], and hands [22,23]. Outcome measures that can be used while managing individuals with distorted body perception are available, including the Fremantle Knee Awareness Questionnaire (FreKAQ). This is a self-reported nine-item questionnaire with a five-point Likert scale, that was developed to assess changes in knee joint’s awareness [24].

FreKAQ is based on a questionnaire originally used for people with chronic low back pain [25]. Higher scores, up to 36, are indicative of lack of body awareness. The questionnaire items are categorised into three independent, yet correlated, dimensions: neglect-like symptoms (items 1−3), decreased proprioceptive acuity (items 4 and 5), and distorted body image (items 7−9), with item 6 assigned to either the second or third dimension [25,26]. The psychometric properties of FreKAQ were tested on individuals with knee OA from Japan. It achieved good internal consistency (Cronbach’s alpha = 0.88) and test-retest reliability (ICC_2,1_ = 0.76 [95% CI = 0.52–0.89]) [24]. In this study, the questionnaire was found to be unidimensional which indicates that its three dimensions represent one construct. It also significantly correlated with, pain intensity, kinesophobia, anxiety, and pain catastrophising.

Since its development, FreKAQ has been adapted to Italian [26] and Persian [27] languages, but no adaptation to Arabic is available. Despite the high prevalence of knee OA in this population, there are limited knee-specific patient-reported outcome measures (PROMs) which might compromise healthcare equality, diversity, and inclusion. Adaptation of FreKAQ into Arabic ensures that this patient population receives assessments that consider their healthcare needs in their own cultural context. Therefore, the purpose of this study was to cross-culturally adapt the FreKAQ into Arabic and to assess the psychometric properties of the adapted version, including its internal consistency, test-retest reliability, convergent validity, discriminant validity, and structural validity.

## Methods

The first part of the study was the adaptation phase which was conducted according to the American Association of Orthopaedic Surgeons guidelines for translation and cultural adaptation of self-report outcome measures [28]. The second part of the study is a cross-sectional study design, consisting of reliability and validity studies to establish the psychometric properties of the adapted version. Permission to use and to adapt FreKAQ has been granted by its developers prior to starting the study. FreKAQ is available via an open access publication [24].

### Ethics statement

The study was given an ethics approval from the Institutional Review Board at University of Jordan Hospital (Ref: 262/2022). Potential participants were given an information sheet detailing the study’s aim and procedures and how their data would be processed before giving their consent. A tick box was added to confirm consent before completing the electronic survey. The survey was active between 01/10/2022− 20/02/2023. The collected data were kept confidential on a secure online server. Only accessible the first author. An anonymised offline version was for data analysis. Personal contact details were removed. Incomplete responses were removed from analysis.

### Study one: Translation and cross‑cultural adaptation study

Stage I (Initial Translation): Independent translation of the English version of FreKAQ [24] into Arabic was conducted by two professional translators, one with a medical background. This step led to production of T1 and T2 versions of FreKAQ. Stage II (Synthesis of the translations: T12 version was then synthesised by a bilingual physiotherapist through discussions with the translators to reach consensus. Stage III (Back translation): Independent translation of T12 version back to English language was conducted by two bilingual physiotherapists, producing BT1 and BT2 versions. Both physiotherapists were naive to FreKAQ and were not familiar with its items before the study. Stage IV (Expert Committee): All translations (original, T1, T2, and T12) were discussed by a committee formed from the first two authors, translators and three individuals with chronic knee pain. The discussion aimed at ensuring clarity and suitability of the items, ensuring appropriate grammatical structure and reaching consensus on any identified discrepancies. The final approved version FreKAQ-A (Appendix 1) was used in the reliability and validity studies.

### Study two: Psychometric properties

#### Reliability study.

The reliability of FreKAQ-A was tested by determining the internal consistency through Cronbach’s alpha and test-retest reliability through Intraclass Correlation Coefficient (ICC), two-way random effects model (ICC_2,1_). Cronbach’s alpha Cut-off value of ≥ 0.7 was considered high-level internal consistency [29]. ICC_2,1_ value greater than 0.75 was considered good to excellent reliability [30]. Item-total correlation was calculated. A value above 0.3 was considered acceptable [31]. The Minimal Detectable Change (MDC) and the standard error of measurement (SEM) were also calculated to determine the extent of test scores’ accuracy. The MDC95% was calculated using the formula MDC95%= $SEM\;X\;1.96\;X\;\sqrt{2}$ [32]. SEM was calculated using the formula: SEM = $SD\;pooled\;X\;.\sqrt{1-ICC\;}$.

#### Validity study.

To test the convergent and discriminant validity of FreKAQ-A, the study used valid and reliable Arabic versions of Pain Catastrophizing Scale (PCS) [33], EQ-5D [34], Central Sensitization Inventory (CSI) [35], and the Chronic Pain Self-efficacy Scale (CPSS) [36]. PCS is a 13-items, five-point Likert scale that explores the presence of catastrophising thoughts or behaviours in people with chronic pain [37]. A cut-off value of PCS ≥ 30 was found to indicate the presence of catastrophising thoughts or behaviours [33]. CSI, which consists of 25 items, was used to explore symptoms that are indicative of symptoms of central sensitization, i.e., nociplastic pain. The presence of each symptom was rated on a five-point Likert scale. A cut-off value of ≥ 40 was found to indicate the presence of central sensitisation [38]. CPSS was used to explore participants’ coping strategies and their confidence in managing their pain and performing activities of daily living. The use of PCS, CSI, and CPSS was expected to capture the relationship between disturbances in knee perception and role of the central nervous system in maintaining pain and disability [39]. EQ-5D was used to assess health related quality of life [40]. It has a visual analogue scale (EQ-VAS) that asks individuals to rate their overall health on a scale from 0 (worst imaginable health) to 100 (best imaginable health), and a descriptive system (EQ-5D-5L) which comprises five domains of mobility, self-care, usual activities, pain/discomfort and anxiety/depression. The outputs of EQ-5D-5L were converted a single index value [34].

***Convergent validity*** was determined by calculating the association between FreKAQ-A and the aforementioned outcome measures. The correlation between FreKAQ-A and scores of PCS and CSI was expected to be positive, whereas its correlation with CPSS and EQ-5D was expected to be negative. The strength of the correlation was interpreted as weak, moderate, and strong if Spearman’s correlation coefﬁcient (*rho*) was 0–0.3, 0.4–0.6, and 0.7–1.0, respectively [41].

***Discriminant validity*** was determined by testing six hypotheses. 1) Individuals with only knee pain would have lower FreKAQ-A mean scores than individuals with additional painful condition. 2) Individuals who underwent knee joint replacement would have higher FreKAQ-A scores. 3) Individuals with PCS total score of 30 or more, i.e., clinically relevant catastrophising [37] would have higher FreKAQ-A scores. 4) Females will have higher FreKAQ-A scores than males. 5) Individuals with BMI of 30 or more would have higher FreKAQ-A. 6) Individuals with CSI total score of 40 or more, i.e., clinically relevant sensitization would have higher FreKAQ-A. Validity was considered good if ≥ 75% of the hypotheses were met. To test these hypotheses the independent samples T test was used. Data were analysed using IBM SPSS (version 25).

***Ceiling or floor effects*** were deemed present if ≥ 15% of participants achieved the highest (36) or lowest (0) possible scores of FreKAQ-A. [42,43]

### Participants

Adult persons who attended appointments at orthopaedic clinics due to osteoarthritis-related knee pain were invited to take part in the study. The clinics were based in one of the major hospitals in Jordan serving patients from various regions both within and beyond the capital, Amman. Participants were excluded if they had poor command of Arabic language; had cognitive or communication problems preventing them from completing the questionnaire; had psychological or neurological problems that compromise sensations. Participants were approached by a research assistant who ensured they met the inclusion criteria: Over the age of 18 years; Arabic speaking; and diagnosed with knee osteoarthritis at least in one joint. The criteria for diagnosis used at the hospital was followed. Upon their consent, participants were asked to complete a web-based survey that asked for participants’ demographics, educational level, location of knee pain, duration of complaint, intensity of current, average, best and worst pain using numerical rating scale NRS, course of pain, surgical replacement of a knee joint (if they had one), date of joint replacement, additional chronic pain complaints, and the outcome measures stated above. A paper-based copy was offered for participants who had difficulty in completing the survey online. For validity study, the total sample size calculated for detecting a significant correlation coefficient of at 0.3 (α = 0.05, power = 0.90) was 120. For the test-retest reliability study, it is suggested that a sample size of 50 participants is adequate [43]. Therefore, with the anticipation of a minimum of 40% response rate between the two rounds of measurements, the study aimed to recruit at least 125 participants.

## Results

### Translation and cross‑cultural adaptation study

During stage IV, the item which was discussed the most was item 6 as the patient group found it difficult to understand the literal translation of the word contour. This was resolved by reverting to the phrase in the original chronic low back pain version of the questionnaire [25]. The word was translated to mean borders or outline of the knee. To establish the face and content validity of the translated questionnaire, 30 persons with knee OA were asked to complete the questionnaire to review the clarity and suitability for the pre-final version’s items. No additional issues were raised, and thus the face and content validity of the questionnaire was confirmed.

### Study two: Psychometric properties

A total of 197 individuals (19.7% males) with osteoarthritic knee pain completed the first round of measurement. All participants completed the online version. Almost 60% had pain in both knees. Their mean age was 61.89 (SD = 11.66; 95% CI = 60.21–63.58). Only 12 individuals (6.1%) had an acute pain of less than three months. Mean weight and height were 85.039 (SD = 14.27; 95% CI = 82.98–87.10), and 162.52 cm (SD = 8.21; 95% CI = 161.33–163.70), respectively. The remaining characteristics of participants are reported in table 1. The mean FreKAQ-A score was 14.88 (SD = 7.58; 95% CI = 13.81–15.94).

**Table 1:** Participants Demographics and Clinical Data (N = 197).

Variable (Range)	N (%)
Sex	
Female	159 (80.7%)
Male	38 (19.3%)
Educational level	
Primary	26 (13.2%)
Middle	25 (12.7%)
Secondary	59 (29.9%)
Graduate	50 (25.4%)
Postgraduate	11 (5.6%)
Otherwise	26 (13.2%)
Location of knee pain	
Right side	38 (19.3%)
Left side	42 (21.3%)
Both sides	117 (59.4%)
Chronicity (Duration of pain)	
< 3 Months	12 (6.1%)
3Months - 1 Year	37 (18.8%)
2–5 Years	67 (34.0%)
6–10 years	39 (19.8%)
> 10 years	42 (21.3%)
Course of pain	
Persistent pain with slight fluctuations	62 (31.5%)
Persistent pain with pain attacks	46 (23.4%)
Pain attacks without pain between them	51 (25.9%)
Persistent pain with short periods of relief	38 (19.3%)
Surgical replacement of the joint	
No	127 (64.5%)
Right side	35 (17.8%)
Left side	31 (15.7%)
Both sides	4 (2.0%)
Date of joint replacement	
< 3 Months	22 (11.2%)
3 Months- 1 Year	24 (12.2%)
2-5 years	20 (10.2%)
6-10 years	1 (.5%)
> 10 years	3 (1.5%)
Additional chronic pain complaints	
No	44 (22.3%)
Yes*	153 (77.7%)
*Headache, migraine, or orofacial pain*	20 (13.1%)
*Post-surgical pain*	16 (10.5%)
*Pain associated with nerve injuries*	5 (3.3%)
*Pain caused by cancer or its treatment*	5 (3.3%)
*Rheumatic pain*	75 (49.0%)
*Fibromyalgia*	7 (4.6%)
*Visceral pain (abdomen or chest)*	10 (6.5%)
*Back pain*	99 (64.7%)
*Neck pain*	61 (39.9%)
*Other complaints, e.g., local foot pain*	14 (9.2%)
BMI (kg/m^2^)	186 (94.4%)**
Normal	14 (7.1%)
Overweight	50 (25.4%)
Obese class I (BMI of 30 to < 35)	66 (33.5%)
Obese class II (BMI of 35 to < 40)	41 (20.8%)
Obese class III (BMI of 40 or higher)	15 (7.6%)
CSI	
< 40	62 (31.47%)
≥ 40	135 (68.53%)
PCS	
< 30	136 (69.04%)
≥ 30	61 (30.96%)

* Some patients reported more than one complaint.

** Data of 11 individuals were missing.

CSI, central sensitization inventory; PCS, pain catastrophizing scale; BMI, body mass index.

### Reliability

The internal consistency of FreKAQ-A was excellent (Cronbach’s α = 0.87). Cronbach α if the item deleted ranged from 0.851–0.876. Item mean, corrected item-total correlation, and scale mean if item deleted are reported in table 2. All values of corrected item-total correlation were above the cut-off point of 0.30. Seventy individuals, of which 53 were females, participated in the second round. Mean (SD) for current pain = 6.51 (2.36); for best pain = 4.8 (2.49); for worst pain = 8.09 (2.41); for age = 58.5 (11.5); for FreKAQ-A scores = 13.33 (7.34). Differences in any pain parameters between the two rounds were not identified. Test-retest reliability was excellent (ICC_2,1_ = 0.841, 95%CI = 0.745–0.901; p < 0.001). It ranged between 0.46 and 0.88 for individual items (table 2). SEM and MDC_95%_ were 2.92 and 8.09, respectively.

**Table 2:** Outcome of reliability analysis of FreKAQ-A.

Item	Round1 (N = 197)				ICC (95%CI Lower-Upper Bounds) p < 0.001
	Item Mean (SD)	CITC	Scale Mean if Item Deleted	α if item deleted	
1.	1.72 (1.22)	.646	13.16	.856	.799 (.676−.875)
2.	2.18 (1.11)	.638	12.70	.858	.458 (.137−.661)
3.	1.27 (1.17)	.578	13.61	.863	.518 (.221−.701)
4.	1.60 (1.13)	.675	13.28	.854	.500 (.193−.690)
5.	1.70 (1.13)	.716	13.18	.851	.592 (.341−.747)
6.	1.68 (1.16)	.712	13.20	.851	.773 (.635−.859)
7.	2.02 (1.36)	.588	12.86	.863	.880 (.806−.925)
8.	.72 (1.06)	.412	14.16	.876	.684 (.494−.803)
9.	1.99 (1.38)	.566	12.88	.865	.772 (.635−.858)

CITC: Corrected Item-Total Correlation: Measurement of correlation between an individual item and the total score of the scale, excluding that item. Value identifies weak items that does not align with the scale.

Scale Mean if Item Deleted: Measurement of average total score of a scale if a particular item is removed. Value identifies influential items and help in ensuring that each item has meaningful contribution to the scale.

α if item deleted: re-calculation of Cronbach’s alpha coefficient when corresponding item is removed from the scale. Value identifies each item contribution and capture any problematic items if α decreased significantly.

### Validity

The correlations between FreKAQ-A and the study variables are reported in table 3. The strongest association was with PCS (*rho *= 0.71) and EQ-5D-5L (*rho *= 0.62). Weak to moderate association was identified for the remaining variables.

**Table 3:** Construct Validity of FreKAQ-A: Correlation with Related Measures.

Variable (Range)	Mean (SD)/Median	*Rho*
Chronicity (Duration of pain)	2-5 years	0.223*
Current pain intensity (1–10)	6.85 (2.08)	0.438**
Average pain intensity in weeks (1–10)	7.03 (2.22)	0.439**
Best pain in 4 weeks (0–10)	5.12 (2.36)	0.320**
Worst pain in 4 weeks (2–10)	8.28 (1.87)	0.400**
Age	62.25 (11.54)	0.112
Height	162.63 (8.23)	−0.147*
Weight	85.13 (13.93)	0.133
BMI (kg/m^2^)	32.38 (5.37)	0.218**
EQ-VAS^	52.87 (29.15)	−0.249**
EQ-5D-5L index value	0.358 (0.440)	−0.620**
CSI (6–86)	47.08 (15.29)	0.346**
CPSS	108.87 (45.00)	−0.491**
-Pain	24.31 (9.73)	−0.351**
-Function	42.62 (23.59)	−0.487**
-Coping	41.93 (16.84)	−0.445**
PCS	22.32 (12.23)	0.707**
-Helplessness	9.90 (5.84)	0.686**
-Magnification	4.62 (3.26)	0.630**
-Rumination	7.80 (4.02)	0.650**

* Significant at P < .05.

** Significant at P < .001

^Score of 100 indicate best imaginable health status.

CSI, central sensitization inventory; PCS, pain catastrophizing scale; CPSS, chronic pain self-efficacy scale; BMI, body mass index.

Five out of six hypotheses (83%) for discriminant validity were confirmed. One) individuals with only knee pain had significantly lower FreKAQ-A mean scores than individuals who had an additional painful condition, t = −2.863 (195), P < .01. Two) There was no significant difference in FreKAQ-A scores between individuals who had their joint replaced and those who had not, t = 1.67 (160), P = .097. Three) Individuals with clinically relevant pain catastrophizing had significantly higher FreKAQ-A mean scores, t = 10.32 (195), P < .001. Four) Females had significantly higher FreKAQ-A mean scores than males, t = 3.330 (214), P < .001. Five) Individuals with BMI ≥ 30 had significantly higher FreKAQ-A mean scores, t (184) = 2.558, P < .05. Six) Individuals with clinically relevant central sensitization (CSI ≥ 40) had significantly higher FreKAQ-A mean scores, t = 4.23 (195), P < .001.

### Ceiling and floor effect

A maximum score of 32 out of 36 was recorded by only one participant, suggesting no ceiling effect [42]. A minimum score of zero was recorded by 9 participants (4.57%). As shown in table 4, the responses for each item of the FreKAQ-A showed clear floor effect for items three (36%) and eight (62%), with another 6 items having a floor effect ranging from 19.3% to 25.4%.

**Table 4:** Responses for each item of the FreKAQ-A (N = 197).

Item	Never N (%)	Rarely N (%)	Occasionally N (%)	Often N (%)	Always N (%)	Median	Mean
1.I feel my knee is not part of the rest of my body	50 (25.4)	21 (10.7)	70 (35.5)	46 (23.4)	10 (5.1)	2	1.72
2.I need to focus all my attention on my knee to move it in the way I want	20 (10.2)	26 (13.2)	69 (35.0)	62 (31.5)	20 (10.2)	2	2.18
3.I sometimes feel my knee moves involuntary without my own control	71 (36.0)	39 (19.8)	57 (28.9)	23 (11.7)	7 (3.6)	1	1.27
4.When I do my daily activities, I do not know how much my knee moves	43 (21.8)	43 (21.8)	70 (35.5)	32 (16.2)	9 (4.6)	2	1.60
5.When I do my daily activities, I am not sure exactly the position of my knee	38 (19.3)	40 (20.3)	73 (37.1)	36 (18.3)	10 (5.1)	2	1.70
6.I cannot imagine accurately the borders of my knee	45 (22.8)	32 (16.2)	67 (34.0)	47 (23.9)	6 (3.0)	2	1.68
7.My knee appears to become enlarged (swollen)	44 (22.3)	20 (10.2)	48 (24.4)	58 (29.4)	27 (13.7)	2	2.02
8.My knee appears to become atrophied	122 (61.9)	31 (15.7)	25 (12.7)	16 (8.1)	3 (1.5)	0	0.72
9.I feel my knee is not like the other knee (different)	41 (20.8)	29 (14.7)	51 (25.9)	42 (21.3)	34 (17.3)	2	1.99

## Discussion

The study aimed to translate and adapt the FreKAQ into Arabic, and to evaluate the psychometric properties of the adapted version (FreKAQ-A) on individuals with osteoarthritic knee pain. The process of developing the translated version followed the robust guidelines for cross-cultural adaptation of self-report measures [28]. FreKAQ-A had excellent internal consistency and test-retest reliability. Its convergent validity was satisfactory as evidenced by the moderate to strong association with CSI, pain intensity, CPSS, EQ-5D-5L, and PCS. It also had satisfactory discriminant validity as evidenced by fulfilling five out of six of the priori hypotheses. The findings therefore support the use of FreKAQ-A in assessing individuals with osteoarthritic knee pain.

### Reliability

The internal consistency of the FreKAQ-A (Cronbach’s α) was excellent and comparable to the Japanese version (α = 0.88). It was slightly higher than both the Italian (α = 0.74) and Persian (α = 0.73) versions. Test-retest reliability was excellent as with other studies. It was however higher than the ICC reported in the Japanese version (ICC = 0.76), in which items 1, 4, and 5 had an ICC of less than 0.40. All items in the current study had an ICC of more than 0.40 (Table 2). The SEM and MDC95% were the highest in this study possibly due to the diverse background of participants. For clinical use, a change of at least 8 points should be present to attribute any changes to the effect of treatment. Finally, item 8 was not endorsed by more than half of participants (61.9%). The Japanese version showed a similar tendency, but the percentage of endorsement was 47.7%. The remaining two studies did not report the frequency of response for each item.

### Convergent validity: Association with pain outcome measures

The study is the first to explore the association of FreKAQ scores with CSI and CPSS. For both scales, the association was moderate. While the CSI measures disturbances in central nervous system function that are associated with nociplastic pain, CPSS measures the confidence in recovery and coping strategies [38,39]. There is evidence to suggest that pain self-efficacy is lowest in individuals with knee joint swelling that is not confirmed objectively when compared with those who had both subjective and objective swelling [4]. Altogether, these findings support the potential involvement of central nervous system in causing or maintaining perceptual knee disturbances.

The association between FreKAQ-A and psychological variables such as pain catastrophising and stress was identified from the findings of the study. The high level of correlation with pain catastrophising scale was close to the one reported in the Japanese validation of FreKAQ (rho = 0.70) [24], and higher than the correlation reported in the Italian validation (rho = 0.47) [26]. Pain catastrophising is highest in individuals who report knee joint swelling that is not confirmed objectively [4]. It might be argued that perceptual knee disturbances might lead to distress and magnification of threat. However, there is limited evidence to suggest whether problems with knee awareness contribute to increased catastrophizing or if it is the opposite [6,23,44].

The correlation between FreKAQ-A and pain intensity was positive and moderate. This is consistent with the association identified in the Japanese [24] and Italian [26] validation studies (rho = 0.37 and 0.35, respectively). It is also similar to the association identified for the back. [25,45,46] and neck [47] versions of the questionnaire. Disturbances in knee joint awareness can influence the effectiveness of physiotherapy in resolving pain [6]. There is also evidence suggesting that visual and tactile illusion techniques to restore the awareness of knee joint can indeed decrease pain [7,8]. The positive and moderate association between pain intensity and perceptual disturbances might be mediated by other variables such as perceived quality of life, self-efficacy, and pain catastrophising. The mediating role of these variables might be considered in future research. Experimental and longitudinal research designs using interventions such as cognitive behavioural therapy and self-management education programmes can be used to evaluate changes in joint perception as primary outcome. The positive impact of these interventions on reducing pain catastrophising and self-efficacy is established in multiple studies in the context of knee osteoarthritis [48,49,50].

### Discriminant validity: hypotheses testing

The hypothesis that individuals who underwent knee joint replacement would have higher FreKAQ-A scores was not confirmed. Previous studies that validated FreKAQ excluded patients who had knee joint arthroplasty from their validation studies [24,26,27]. This group of patients was included since disturbances in knee joint perception after the surgery is reported [50]. Their data suggest that there is no statistically significant difference in FreKAQ-A scores between them and those who did not perform knee arthroplasty. The value of this finding can be explored further. Anecdotal notes suggest that some patients attribute these disturbances in perception to a presumable failure of the joint replacement surgery which might augment their levels of stress and anxiety. Those individuals might be assured that the abnormalities in the joint’s perception is not linked to the position of the implant or failed operation. Rather, it might be linked to other factors such as changed neural activity, pain catastrophising, and increased levels of stress and anxiety.

The association between FKAQ-A and duration of pain was weak (*rho* = 0.223, P < .05). In the Japanese language adaptation, the association was not significant, *rho* = −0.06, P = 0.76 [24]. This discrepancy in the strength of association is evident in the back version where some studies identified no significant association [45,46,51,52], while others identified weak to high association [25,53,54]. It is suggested that with longer duration of pain, more cortical changes that lead to higher perceptual disturbances would be present [12,44]. Reasons for variabilities in association in these studies could be linked to the chronicity of condition, or to the unit of measuring the duration of suffering, i.e., years or months. Nonetheless, disturbances in joint awareness should not be solely attributed to the duration of suffering.

### Strengths and limitations

One of the strengths of this study is the inclusion of individuals suffering from chronic knee pain in the first stage of the study. Their contribution ensured that the scale is complete and comprehensible before moving to the reliability and validity studies. Also, having seventy participants for the reliability study fulfilled the recommended sample size. On the other hand, the study has some limitations [43]. First, it did not include a healthy control group. While the study identified the ability of FreKAQ-A to discriminate between certain groups of individuals with osteoarthritic knee pain, the inclusion of a control group could have eliminated any bias in interpreting the scale’s items [55]. Second, participants were not instructed to stop any treatment during the reliability study. However, It is less likely that some symptoms related to body awareness have changed during the 10–14 days gap [56]. Third, the under-representation of male participants (only 19.7% of total sample) can affect generalisability of findings. Further studies with a balanced sample can provide robust psychometric measurements. Finally, it is evident that floor effect existed for scoring 8 out of the 9 items. Considering items on the scale, variability in responses would be expected as some items naturally fall on opposite ends of the scale, e.g., enlarged vs. shrunken knee. However, it is less likely that this would limit the use of the scale as the sum of all items did not show evidence of a floor effect.

## Conclusion

The study has adapted Fremantle Knee Awareness Questionnaire into Arabic. The adapted version showed excellent reliability and internal consistency. It has satisfactory validity that is consistent with similar studies. Considering the growing number of Arabic speaking individuals with knee OA, the use of the questionnaire in clinical settings can provide clinicians with insights about potential factors that maintain knee pain. Certainly, it is not a substitute for other clinical outcome measures, but it can guide clinical decision-making by choosing treatment approaches that target impairments of proprioceptive acuity or knee awareness when identified.

## Supporting information

S1 Appendix FKAQArabic Scale. Appendix 1. The Arabic Fremantle Knee Awareness Questionnaire FreKAQ-A. Appendix 2. The Fremantle Knee Awareness Questionnaire. (PDF)

S1 File The Fremantle Knee Awareness Questionnaire. (DOCX)
