# Correction: Translation, cross-cultural adaptation and psychometric properties of the Arabic version of the Fremantle Knee Awareness Questionnaire in people with knee osteoarthritis

**DOI:** 10.1371/journal.pone.0339036

**Published:** 2025-12-15

**Authors:** Mohammad Madi, Hayat Hamzeh, Sumayeh Abujaber, Ibrahim Altubasi

The Funding statement for this article is incorrect. The correct Funding statement is as follows: This study was financially supported by the Deanship of Scientific Research at the University of Jordan: https://research.ju.edu.jo/home.aspx.
