# Supplementary material for: Translation, cross-cultural adaptation and psychometric properties of the Arabic version of the Fremantle Knee Awareness Questionnaire in people with knee osteoarthritis
Source: PLoS One. 2025 Jul 15;20(7):e0328228. doi: 10.1371/journal.pone.0328228 (PMC12262845; doi:10.1371/journal.pone.0328228)
Supplement: S1 Appendix — (PDF) [file pone.0328228.s001.pdf]

استبيان فريمانتل لقياس درجة الوعي بالركبة

فيما يلي بعض الأشياء التي أخبرنا بها أشخاص يعانون من آلام الركبة عن شعورهم تجاه ركبتهم. باستخدام المقياس التالي ، يرجى الإشارة إلى الدرجة التي تشعر بها **بأي من التالي عندما تعاني من آلام الركبة**:

0: **لا تشعر** هكذا **مطلقاً**
1: **نادراً ما** تشعر هكذا
2: أحياناً أو في بعض الأحيان يبدو تشعر هكذا
3: **غالباً** أو قدر معتدل من الوقت تشعر هكذا
4: **دائماً** أو معظم الوقت تشعر هكذا

	**مطلقاً**	**نادراً**	أحياناً	**غالباً**	**دائماً**
1. اشعر كأن ركبتي ليست جزءاً من باقي جسدي.	0	1	2	3	4
2.احتاج الى تركيز كل انتباهي على ركبتي لاجعلها تتحرك بالطريقة التي أريدها.	0	1	2	3	4
3. اشعر احيانا بان ركبتي تتحرك بشكل لاارادي بدون تحكم مني.	0	1	2	3	4
4. عند أداء مهامي اليومية لا أعرف المقدار الذي تتحرك به ركبتي.	0	1	2	3	4
5. عند أداء مهماتي اليومية لست متأكداً من وضعية ركبتي على وجه التحديد.	0	1	2	3	4
6.لا استطيع تصور حدود ركبتي بدقة.	0	1	2	3	4
7.تبدو ركبتي وكأنها تضخمت (انتفخت).	0	1	2	3	4
8. تبدو ركبتي وكأنها تقلصت.	0	1	2	3	4
9. اشعر أن ركبتي غير مماثلة للركبة الاخرى (أو مختلفة).	0	1	2	3	4
