# Supplementary material for: Translation, cross-cultural adaptation and psychometric properties of the Arabic version of the Fremantle Knee Awareness Questionnaire in people with knee osteoarthritis
Source: PLoS One. 2025 Jul 15;20(7):e0328228. doi: 10.1371/journal.pone.0328228 (PMC12262845; doi:10.1371/journal.pone.0328228)
Supplement: S1 File — (DOCX) [file pone.0328228.s002.docx]

***The Fremantle Knee Awareness Questionnaire***

Here are some things that other people with low knee pain have told us about how their knee feels to them. Using the following scale, please indicate the degree to which your knee feels this way **when you are experiencing knee pain**

**0 = Never feels like that**

**1 = Rarely feels like that**

**2 = Occasionally, or some of the time feels like that**

**3 = Often, or a moderate amount of time feels like that**

**4 = Always, or most of the time feels like that**

| **Never** | **Rarely** | **Occasionally** | **Often** | **Always** |  |
| --- | --- | --- | --- | --- | --- |
| 1. My knee feels as though it is not part of the rest of my body | 0 | 1 | 2 | 3 | 4 |
| 1. I need to focus all my attention on my knee to make it move the way I want it to | 0 | 1 | 2 | 3 | 4 |
| 1. I feel as if my knee sometimes moves involuntarily, without my control | 0 | 1 | 2 | 3 | 4 |
| 1. When performing everyday tasks, I **don’t** know how much my knee is moving | 0 | 1 | 2 | 3 | 4 |
| 1. When performing everyday tasks, I am **not** sure exactly what position my knee is in | 0 | 1 | 2 | 3 | 4 |
| 1. I **can’t** perceive the exact outline of my knee | 0 | 1 | 2 | 3 | 4 |
| 1. My knee feels like it is enlarged (swollen) | 0 | 1 | 2 | 3 | 4 |
| 1. My knee feels like it has shrunk | 0 | 1 | 2 | 3 | 4 |
| 1. My knee feels lopsided (asymmetrical) | 0 | 1 | 2 | 3 | 4 |

Available as an open access publication:

Nishigami T, Mibu A, Tanaka K, Yamashita Y, Yamada E, Wand BM, et al. Development and psychometric properties of knee-specific body-perception questionnaire in people with knee osteoarthritis: The Fremantle Knee Awareness Questionnaire. PloS one. 2017;12(6):e0179225.
